# Characterizing tuberculosis diagnosis and the associations with economic instability and employment discrimination among women living with HIV across 11 countries in sub‐Saharan Africa: a cross‐sectional study

**DOI:** 10.1002/jia2.70022

**Published:** 2026-01-06

**Authors:** Carrie Lyons, Omar Syarif, Pim Looze, Gnilane Turpin, Jean de Dieu Anoubissi, Sophie Brion, Keren Dunaway, Yi‐Chi Chiu, Daria Ocheret, Laurel Sprague, Carlos Garcia de Leon Moreno, Hatim Sati, Amrita Rao, Katherine Rucinski, Stefan Baral, Richard Chaisson, David Dowdy, Chris Beyrer, Becky Genberg

**Affiliations:** ^1^ Johns Hopkins School of Public Health, Epidemiology Baltimore Maryland USA; ^2^ Global network of People living with HIV (GNP+) Amsterdam Netherlands; ^3^ The International Community of Women Living with HIV (ICW) Johannesburg South Africa; ^4^ UNAIDS Geneva Switzerland; ^5^ World Health Organization Geneva Switzerland; ^6^ Johns Hopkins School of Public Health, International Health Baltimore Maryland USA; ^7^ Johns Hopkins School of Medicine Baltimore Maryland USA; ^8^ Duke Global Health Institute Durham North Carolina USA

**Keywords:** discrimination, employment, HIV, policy, tuberculosis, women

## Abstract

**Introduction:**

Tuberculosis (TB) is the leading cause of death among people living with HIV. Global estimates among people living with HIV demonstrate that more incident cases and more deaths due to TB occur among women than men. Simultaneously, women experience higher levels of under and unpaid work compared to men. Given that poverty is an established determinant for TB, the aim of this study is to characterize the role of HIV‐related employment discrimination and legal protections on TB outcomes for women living with HIV.

**Methods:**

The People Living with HIV Stigma Index 2.0 study was implemented in 11 countries across sub‐Saharan Africa, including Angola, Benin, Burkina Faso, Cote D'Ivoire, Ghana, Kenya, Mauritania, Nigeria, Lesotho, Togo and Zimbabwe. Study design and implementation were led by networks of people living with HIV in each country between 2020 and 2022. Interviewer‐administered questionnaires were used to collect self‐reported socio‐behavioural measures among cisgender adult women living with HIV. Multilevel logistic regression models were used to estimate associations between economic instability and employment discrimination exposures and recent TB diagnoses in the context of varying discrimination protections for women living with HIV.

**Results:**

Among 10,718 participants, 7.5% (*n* = 807) reported a recent TB diagnosis. Among women in countries without non‐discrimination protections, recent TB diagnosis was negatively associated with current employment (aOR: 0.72; 95% CI: 0.62, 0.85) compared to no employment; and positively associated with being refused employment or income due to HIV status (aOR: 1.80; 95% CI: 1.36, 2.39) and ever being refused promotion (aOR: 2.00; 95% CI: 1.37, 2.91) compared to those who have not reported these experiences. Among women in countries with non‐discrimination protections, recent TB diagnosis was associated with lower current employment (aOR: 0.72; 95% CI: 0.56, 0.92) but not associated with employment discrimination.

**Conclusions:**

The presence of social protections may modify the associations between employment discrimination and TB diagnosis. Employment discrimination was associated with TB diagnosis in settings without social protections but not in settings with those protections in place—highlighting a potential vulnerability among people living with HIV in settings without non‐discrimination protections. Given the role of poverty in driving TB epidemics, social protections focused on employment, economic instability and opportunity may support TB prevention and control.

## 1 INTRODUCTION

Globally, approximately 40 million people are living with HIV, and there were 630,000 deaths attributable to HIV in 2023 [1]. Tuberculosis (TB) is the leading cause of death among people living with HIV (PLHIV) and disproportionately so in low‐income settings [2]. PLHIV are nearly 20 times more likely to develop active TB than those not living with HIV, and the development of TB disease among PLHIV is associated with suboptimal treatment outcomes and being immunocompromised [3]. Conversely, incidence is lower among PLHIV on antiretroviral therapy (ART) compared to those not on treatment [4]. The scale‐up of ART along with the integration of HIV and TB services has reduced TB‐related deaths among PLHIV by 58% globally [1].

Men have higher TB incidence and mortality compared to women overall [4, 5, 6]; however, patterns in sex differences of TB outcomes among PLHIV vary [4, 5]. Recently, among PLHIV, women experienced more incident cases of TB and more TB‐related deaths than men [5]. In sub‐Saharan Africa (SAA), two‐thirds of new HIV acquisitions in 2023 were among women, and the burden of TB among young women in this region is increasing [1, 7]. Given the observed sex differences in TB outcomes, which may vary based on HIV status, examining factors associated with TB among women living with HIV separately from men may provide a better understanding of the specific risks for TB among women living with HIV.

Poverty and socio‐economic status are established determinants of TB risk for both acquisition and mortality [2, 8]. Development of TB disease among individuals living with HIV is also influenced by social and economic determinants, such as housing, employment, and access to affordable and quality healthcare [9, 10, 11, 12, 13]. In resource‐limited settings, women are disproportionately affected by economic instability. Currently, 41% of women in SSA are living below their respective country's poverty line [14]. Women are often affected by lower wages and higher levels of unpaid care work than their male counterparts, and this gap increased during the COVID‐19 pandemic [15, 16]. Economic instability among women may be influenced in part by discrimination affecting employment, and women living with HIV may experience compounded barriers to economic opportunities due to both gender and HIV status. Employment discrimination among PLHIV, including forced disclosure of HIV status, exclusion in the workplace and loss of employment, is prevalent across settings [17]. A multicounty assessment found that people affected by TB reported employment discrimination as well [18]. Employment discrimination has been shown to be associated with poor health outcomes among women, including depression and anxiety [19, 20]. However, there is a need to better understand the relationship between employment discrimination and TB outcomes, particularly considering the impact of economic stability on TB risk and progression.

Non‐discrimination is a core principle of the Universal Declaration of Human Rights, and aims to prohibit distinction, exclusion, restriction or preference which would impede one's equal rights [21]. Non‐discrimination protections include the establishment and enforcement of national policies to ensure these protections, and is an approach to support equal access to health, economic opportunities and wellbeing. The World Health Organization (WHO) and the Joint United Nations Programme on HIV/AIDS (UNAIDS) have prioritized the adoption of policies for non‐discrimination protections as a structural approach for HIV and TB control [22, 23, 24]. Although non‐discrimination protections is a key strategy to improve HIV and TB epidemics, analyses of the impact of rights protections related to HIV to date have been limited [25, 26]. In some cases, such as in South Africa, a reduction in human rights violations was not observed alongside protective laws [27]. However, studies have shown improvements in HIV prevention, treatment and engagement in care alongside non‐discrimination protections in settings such as Brazil, South Africa, Taiwan and Thailand [26, 28, 29]. Given the promise of non‐discrimination protections for HIV outcomes, we expect these protections may also influence TB outcomes among PLHIV. However, assessments on the impact and role of social protections, specifically non‐discrimination protections, in mitigating risks for TB across countries remain limited.

The primary aim of this study is to assess the associations of economic instability and employment discrimination with recent TB diagnosis and assess if these associations vary based on the presence of national non‐discrimination protection policies.

## 2 METHODS

**Figure 1:**
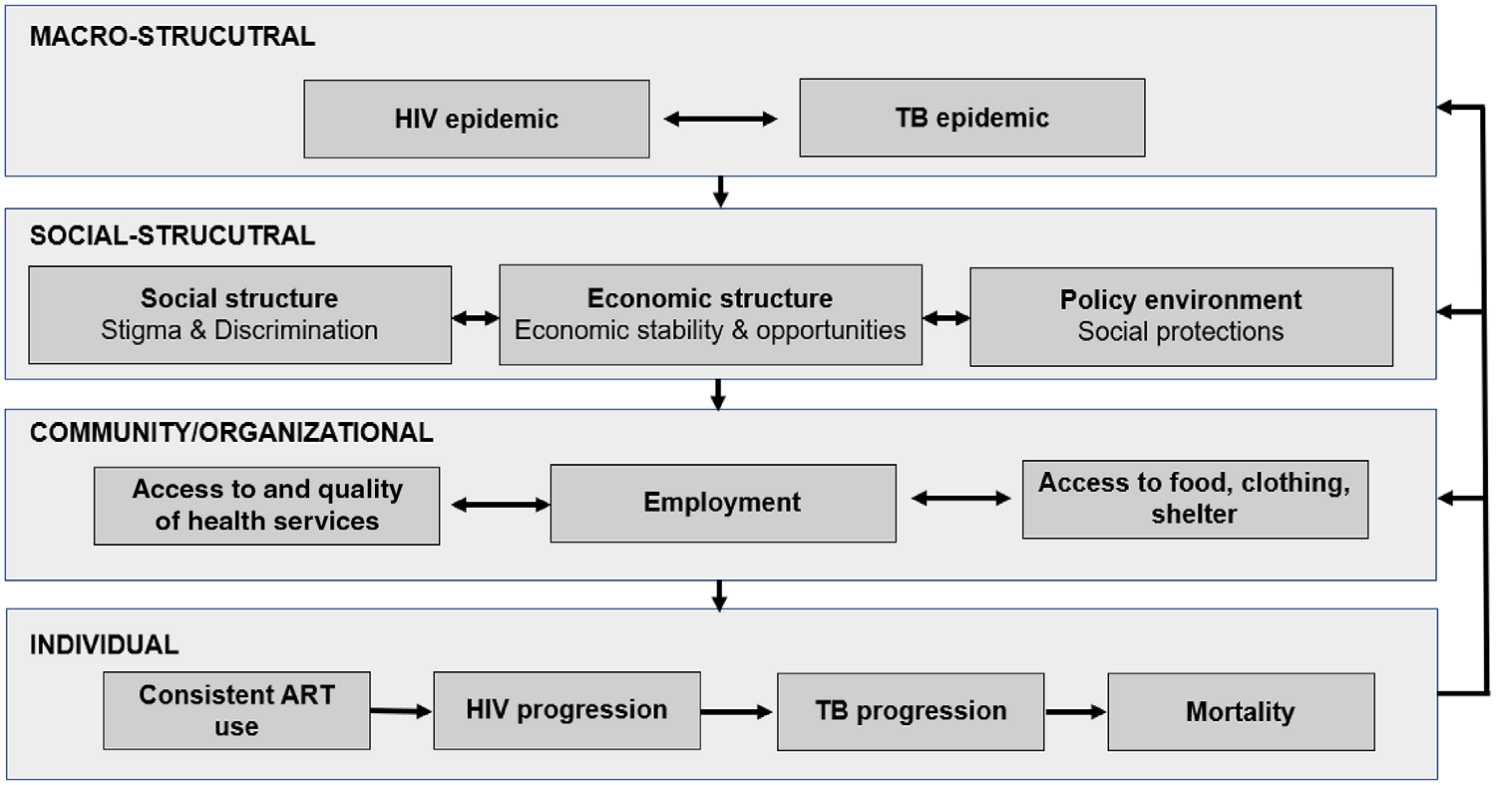
Conceptual model for social and structural drivers of recent TB diagnosis among women living with HIV.

We developed a conceptual model based on existing frameworks to guide research questions and inform our analytical approach (Figure 1) [30, 31, 32]. This model aims to outline risks for the development of TB disease across the individual, community/organizational, social‐structural and macrostructural levels among PLHIV. Additionally, it depicts how drivers of development of TB disease are driven by HIV and TB epidemics, through social and economic structures as well as policy environments; and that these higher‐level factors influence uptake and access to health services, employment, basic needs, and ultimately ART use, development of TB disease and mortality.

### 2.1 Data collection and participants

This multi‐country study used cross‐sectional, individual‐level data collected through the PLHIV Stigma Index 2.0 [33] merged with country‐level data to examine the policy and epidemiologic context. Countries were selected based on having implemented the PLHIV Stigma Index 2.0 study and being in SSA. The PLHIV Stigma Index 2.0 was implemented in each country following a country‐specific protocol, which observed a standardized approach and was approved by the PLHIV Stigma Index 2.0 International Partnership. Data collection in each of the 11 countries was led by networks of PLHIV in partnership with national and international organizations and networks. The PLHIV Stigma Index 2.0 sampling approach combined limited chain referral sampling alongside convenience samples from within venues frequented by PLHIV. However, specific recruitment approaches were designed by the implementation team in each respective country. Data linking sampling approaches to individual participants was not available in the data and, therefore, not included in the analysis.

Survey data were collected between 2020 and 2022, depending on each country‐specific timeline (Table S1). Socio‐behavioural data were collected through interviewer‐administered questionnaires conducted by PLHIV who were trained in survey administration. Inclusion criteria for participation were: ≥18 years of age; aware of HIV‐positive status for >12 months; capable of providing consent; spoke the dominant language; and provided informed consent. The study sample used for this analysis was limited to individuals assigned female sex at birth and identifying as women.

Country‐level data were obtained from several different publicly available sources to characterize the country‐level policy or epidemiologic context. National policies for non‐discrimination protections for each country were obtained from the HIV Policy Lab Database [34]. Country‐level TB incidence, TB treatment coverage and TB funding were obtained from the WHO Global Tuberculosis Programme database [35]. HIV prevalence estimates were obtained from UNAIDS [36].

### 2.2 Measures

Measures were categorized as individual‐level and country‐level variables. Individual‐level variables represent data from self‐reported individual‐level responses from the PLHIV Stigma Index 2.0 survey; and country‐level variables represent aggregate national‐level estimates obtained from HIV Policy Lab, UNAIDS and WHO.

Individual‐level variables described self‐reported characteristics and experiences by women living with HIV who participated in the survey. The primary outcome of interest was recent TB diagnosis. The recent TB diagnosis was determined based on self‐reported diagnosis with TB by a healthcare provider within 12 months prior to survey participation.

Individual exposures of interest included economic instability and employment discrimination based on the conceptual roles and relationships outlined in the conceptual model. Economic instability was defined based on two separate measures: current employment and ability to meet basic needs. Participants were categorized as employed if they reported any employment, regardless of effort or salary level. Being unable to meet basic needs, including food, shelter or clothing, in the last 12 months was categorized with response options as never, sometimes or most of the time. HIV‐related employment discrimination was measured with two separate survey items: the first item was reporting ever having been refused employment or income due to HIV status; the second item was ever having been refused a promotion due to HIV status [37].

Individual‐level potential confounders for our relationships of interest included age, relationship status, educational attainment and years since HIV diagnosis. Relationship status was defined as currently being in an intimate and/or sexual relationship. Educational attainment was defined as any formal education or no formal education. Years since HIV diagnosis is defined as years that the person has known their HIV‐positive status.

Country‐level variables include measures describing country‐level characteristics. Country‐level policies were collected through the HIV Policy Lab [38]. The policy categories were determined based on the year of data collection in each respective country (Table S1). Progress towards implementation of policies was categorized based on adoption of each policy, as “Not adopted” or “Adopted.” Non‐discrimination protections were defined as adopted if national laws protected people from discrimination, including employment discrimination, based on HIV status and gender identity/diversity. The policy categories were determined based on the year of data collection in each respective country.

Country‐level TB incidence was defined as the rate of new cases per 100,000 population in 2021 (Table S2) [35]. TB treatment coverage was estimated as the number of new and relapse cases detected and treated in 2021, divided by the total estimated number of incident TB cases in 2021, expressed as a percentage. Country‐level TB funding was defined as the total funding dedicated towards TB in 2021, including international and domestic funding in millions of US dollars. Country‐level HIV prevalence was defined as the population prevalence of HIV among adults ages 15–49 in each of the respective countries in 2021, based on UNAIDS country estimates [36].

### 2.3 Statistical analysis

We conducted exploratory data analysis to characterize the study population according to recent TB diagnosis. We stratified the sample by recent TB diagnosis and compared the relative frequency of characteristics among women with and without recent TB diagnosis. We calculated the intra‐class correlation coefficient (ICC) to estimate the percentage of the total variance in recent TB diagnosis that reflected variance between countries using an unadjusted, multi‐level mixed effects model with random intercepts.

Our primary exposures of interest were economic instability and employment discrimination. We estimated the association between self‐reported exposures of economic instability and employment discrimination with self‐reported recent TB diagnosis among women living with HIV. These variables were assessed in separate multilevel logistic regression models with random intercepts. We first used unadjusted models with random intercepts; then adjusted models, and each model included individual‐level confounders, including age, education, relationship, time from HIV diagnosis and country‐level confounders, including HIV prevalence; TB incidence, funding and treatment coverage. Potential confounders were included in the models as they were conceptualized as being associated with exposures of interest and a risk factor for the outcome. Statistical significance was defined as the 95% confidence interval (CI) not including the value of 1.00. We then stratified analyses by non‐discrimination policy status.

Effect measure modification (EMM) by policy was evaluated using stratified estimates as well as interaction terms within the regression analysis. Our main assessment of EMM was through stratum‐specific estimates. EMM was determined to be qualitatively present if the direction of the effect estimate changed, or if the 95% CI interval changed by crossing the null. EMM was assessed to be statistically significant if the *p*‐value of the interaction term between policy and our exposure of interest was <0.01.

Given the low ICC, we ran non‐hierarchical logistical regression models as a sensitivity analysis. These are presented in Tables S3 and S4.

The missingness of recent TB diagnosis was examined. Approximately 0.75% were missing responses (*N* = 81). Based on an examination of the correlation matrix between the primary outcomes and other available data, missingness did not follow any observable pattern. Given the low level of missingness in the outcome (<1.0%), and no patterns were observed, complete case analysis (List‐Wise Deletion) was used in all models.

### 2.4 Human subjects research

The protocols for data collection were approved by the ethical committees in each respective country. Approval for secondary data analysis of de‐identified data was provided by the Johns Hopkins Institutional Review Board.

## 3 RESULTS

### 3.1 Study population

Among 10,718 participants, 8.5% (*n* = 915) were in Angola; 5.4% (*n* = 580) in Benin; 15.5% (*n* = 1661) in Burkina Faso; 14.2% (*n* = 1552) in Cote d'Ivoire; 12.3% (*n* = 1316) in Ghana; 11.8% (*n* = 1261) in Kenya; 3.7% (*n* = 396) in Mauritania; 7.6% (*n* = 818) in Lesotho; 7.6% (*n* = 791) in Nigeria; 6.9% (*n* = 737) in Togo; and 6.7% (*n* = 721) in Zimbabwe (Table S1). Among participants, 79.1% (*n* = 8481) were living in countries with non‐discrimination protections based on gender and HIV status (Table 1).

**Table 1:** Distribution of social and structural‐level factors related to economic instability, economic discrimination and social protections for women living with HIV stratified by self‐reported recent TB diagnosis among women living with HIV across 11 countries in sub‐Saharan Africa

	Total^a^		Recent TB diagnosis				
			No		Yes		
	*N*	*%*	*N*	*%*	*N*	*%*	*p*‐values
**Demographic characteristics**							
Age categories							0.269
18−24	899	8.35	823	8.43	62	7.72	
25−30	1581	14.85	1462	14.98	106	13.20	
31+	8175	76.80	7476	76.59	635	79.08	
Currently in a relationship							0.001
No	4445	41.50	4015	40.87	380	47.15	
Yes	6265	58.50	5808	59.13	426	52.85	
Education							0.547
No formal education	2812	26.24	2560	26.05	218	27.01	
Some formal education	7905	73.76	7269	73.95	589	72.99	
**Economic instability**							
Employment							<0.001
No employment	4436	41.39	3981	40.50	402	49.81	
Any employment	6281	58.61	5848	59.50	405	50.19	
Unable to meet basic needs (food, shelter or clothing)							<0.001
Never	2096	19.56	1951	19.85	133	16.48	
Sometimes	5951	55.53	5479	55.75	424	52.54	
Most of the time	2669	24.91	2398	24.40	250	30.98	
**Employment discrimination**							
Ever refused employment or income due to HIV status							<0.001
No	8688	93.30	8000	93.57	610	89.44	
Yes	624	6.70	550	6.43	72	10.56	
Ever refused promotion due to HIV status							<0.001
No	8084	94.86	7448	95.15	560	91.06	
Yes	438	5.14	380	4.85	55	8.94	
**HIV treatment history**							
Consistent ART use							<0.001
No	1694	17.08	1531	16.80	160	21.92	
Yes	8223	82.92	7582	83.20	570	78.08	
**Structural characteristics**							
Non‐discrimination protections							0.921
Not adopted	2237	20.87	5695	57.93	469	58.12	
Adopted	8481	79.13	4135	42.07	338	41.88	

Abbreviations: ART, antiretroviral therapy; TB, tuberculosis.

^a^ Total includes the entire study sample including those who reported the outcome (yes or no) and those with missing data for the outcome.

The median age of participants was 39 years with an interquartile range (IQR) of 31–47; with 8.4% (*n* = 899) between 18–24 years old, and 14.9% (*n* = 1581) between 25–30 years old, and 76.8% (8175) over 30 years old. Overall, 58.5% (*n* = 6265) were in a relationship, and 73.8% (*n* = 7905) completed some formal education.

Among the study population, 0.76% (*n* = 81) did not report recent TB diagnosis status; of the remainder, 7.5% (*n* = 807) reported TB diagnosis within the last 12 months. In this sample, 0.39% of the total variation in the recent TB diagnosis is attributable to differences between countries (ICC = 0.0039).

The median years since HIV diagnosis was 7 (IQR: 3−13). Among participants, 94.3% (*n* = 10,098) reported ever being on ART, and among those, 82.9% (*n* = 8223) reported consistent ART use.

### 3.2 Economic instability and employment discrimination

Among participants, 58.6% (*n* = 6281) reported any type of current employment; and 55.5% (*n* = 5951) and 24.9% (*n* = 2669) reported being sometimes or often unable to meet basic needs, respectively. 6.7% (*n* = 624) reported ever being refused employment or income due to HIV status, and 5.1% (*n* = 438) reported ever being refused a promotion due to HIV status. Recent TB diagnosis was negatively associated with employment (aOR: 0.72; 95% CI: 0.62, 0.85); and positively associated with being unable to meet basic needs often compared to never (aOR: 1.36; 95% CI: 1.07, 1.73). Recent TB diagnosis was also associated with being refused employment or income due to HIV status (aOR: 1.80; 95% CI: 1.36, 2.39) and with being refused promotion due to HIV status (aOR: 1.84; 95% CI: 1.33, 2.55) (Table 2).

**Table 2:** Economic instability, and employment discrimination and the associations with recent TB diagnosis among women living with HIV in 11 countries in sub‐Saharan Africa

	Recent TB diagnosis					
			Unadjusted model		Adjusted for level 1 and 2 variables	
	*N*	%	OR	95% CI	AOR^a^	95% CI
**Economic instability**						
Employment (any)	6281	58.61	0.72	0.62, 0.84	0.72	0.62, 0.85
Unable to meet basic needs (food, shelter or clothing)						
Never	2096	19.56	Ref	Ref	Ref	Ref
Sometimes	5951	55.53	1.12	0.92, 1.37	1.09	0.88, 1.35
Most of the time	2669	24.91	1.44	1.15, 1.79	1.36	1.07, 1.73
**Employment discrimination**						
Ever refused employment or income due to HIV status	624	6.70	1.68	1.28, 2.20	1.80	1.36, 2.39
Ever refused promotion due to HIV status	438	5.14	1.85	1.36, 2.51	1.84	1.33, 2.55

Abbreviations: aOR, adjusted odds ratio; ART, antiretroviral therapy; OR, odds ratio; TB, tuberculosis.

^a^ Adjusted for age, education, relationship, time from HIV diagnosis, current ART use, time from diagnosis to treatment initiation, HIV prevalence among adults, HIV prevalence among women, TB incidence, TB funding and TB treatment coverage.

Among 6244 participants in settings without non‐discrimination protections, 57.5% (*n* = 3591) reported current employment; and 55.6% (*n* = 3473) and 25.0% (*n* = 1562) reported being unable to meet basic needs sometimes and often, respectively. 8.2% (*n* = 444) reported ever being refused employment or income due to HIV status, and 6.6% (*n* = 319) reported ever being refused a promotion due to their HIV status. In settings without non‐discrimination protections, recent TB diagnosis was negatively associated with current employment (aOR: 0.74; 95% CI: 0.60, 0.92); and was positively associated with being refused employment or income due to HIV status (aOR: 1.98; 95% CI: 1.42, 2.77) and ever being refused promotion due to HIV status (aOR: 2.00; 95% CI: 1.37, 2.91) (Table 3).

**Table 3:** Economic instability, and employment discrimination and associations with recent TB diagnosis among women living with HIV in 11 countries in sub‐Saharan Africa stratified by country‐level non‐discrimination protections

	Lack of non‐discrimination protections						Presence of non‐discrimination protections						
	Total (*N*=6244)		Recent TB diagnosis (*N*=469)				Total (*N*=4311)		Recent TB diagnosis (*N*=338)				Interaction^b^
	%	*N*	%	*N*	AOR^a^	95% CI	%	*N*	%	*N*	AOR^a^	95% CI	*p*‐value
**Economic instability**													
Employment (any)	57.52	3591	49.68	233	0.74	0.60, 0.92	60.13	2690	50.89	172	0.72	0.56, 0.92	0.799
Unable to meet basic needs													
Never	19.34	1207	16.84	79	Ref	Ref	19.87	889	15.98	54	Ref	Ref	
Sometimes	55.64	3473	55.44	260	1.18	0.89, 1.56	55.39	2478	48.52	164	0.99	0.70. 1.39	0.562
Most of the time	25.02	1562	27.72	130	1.16	0.83, 1.60	24.74	1107	35.50	120	1.64	1.14, 2.36	0.072
**Employment discrimination**													
Ever refused employment or income due to HIV status	8.16	444	13.72	55	1.98	1.42, 2.77	4.65	180	6.05	17	1.52	0.87, 2.63	0.413
Ever refused promotion due to HIV status	6.57	319	12.50	43	2.00	1.37, 2.91	3.25	119	4.43	12	1.50	0.78, 2.88	0.398

Abbreviations: aOR, adjusted odds ratio; ART, antiretroviral therapy; OR, odds ratio; TB, tuberculosis.

^a^ Multi‐level logistic regression model with random intercepts adjusted for age, education, relationship, time from HIV diagnosis, consistent ART use, HIV prevalence among adults, TB incidence, TB funding and TB treatment coverage.

^b^ We included an interaction term between our exposure of interest and non‐discrimination policy to the model to assess if the association was statistically significantly different between the policy environments.

Among 4311 participants in settings with non‐discrimination protections, 60.1% (*n* = 2690) reported any employment; and 55.4% (*n* = 2478) and 24.7% (*n* = 1107) reported being unable to meet basic needs sometimes and often, respectively. 4.7% (*n* = 180) reported ever being refused employment or income due to HIV status, and 3.3% (*n* = 119) reported ever being refused a promotion due to their HIV status. In settings with non‐discrimination protections, recent TB diagnosis was negatively associated with current employment (aOR: 0.72; 95% CI: 0.56, 0.92). A recent TB diagnosis was positively associated with being unable to meet basic needs, often compared to never (aOR: 1.64; 95% CI: 1.14, 2.36).

## 4 DISCUSSION

Findings from this study suggest that economic instability and employment discrimination were associated with recent TB diagnosis among women living with HIV, and the presence of supportive policies modified these associations. Current employment was associated with lower odds of recent TB diagnosis. We also found that employment discrimination experienced by women living with HIV was associated with higher levels of TB diagnosis within the last year; however, these associations were only observed in settings without non‐discrimination protections. Overall, this study supports that socio‐economic position and opportunity, as well as working conditions, are social determinants of health, including TB [31].

Across the 11 countries studied, current employment was associated with lower odds of recent TB diagnosis. These findings align with existing evidence and the established understanding of the relationship between employment, poverty and risk for TB [2, 8]. Economic instability among women is also associated with food insecurity, experiences of violence and poor mental health outcomes that also contribute to poor outcomes for both TB and HIV [39, 40]. Discouragingly, economic strain and gender inequities have worsened, with women's employment rate declining, and at a greater rate than male counterparts [16]. In our study, the prevalence of employment was marginally higher in settings with non‐discrimination protections; however, the negative association between employment and TB diagnosis was consistent regardless of the non‐discrimination policy setting, suggesting that the existence of policies did not reduce the potential harm of unemployment on TB. This suggests there is a need to better understand the implementation of existing non‐discrimination policies. Existing evidence suggests that limited employment and economic instability may influence the risk for TB development or progression, which supports findings from our study [2, 8]. Conversely, we may be observing these associations because women with recent TB may be unable to work because of disease progression. However, a study in Kenya and Zambia among PLHIV reported that unemployment was largely attributable to discrimination rather than illness [17]. Regardless of the direction of causality, these findings highlight that women living with HIV and TB may be unable to meet their economic needs to support health and wellbeing. Notably, the measure of employment status in this study does not account for the level of income or stability of employment. The informal economy contributes to a large portion of the labour markets in SSA, and informal employment represents 92% of employment among women in the region [41]. Therefore, social protections for employment, benefits or wages may not currently support women in the informal economy, and, therefore, may not adequately support the majority of women in SSA. Social protections and poverty alleviation are key components of the TB response; however, efforts are largely focused on alleviating income loss from TB treatment instead of also supporting overall economic stability [42]. Our findings suggest there is a need for an improved legal framework around social protections for employment and better alignment of social protection frameworks with national HIV and TB response strategies.

Employment discrimination was associated with increased odds of TB diagnosis in settings without non‐discrimination protections, but this association was not observed in settings with these policies. This study suggests that employment discrimination may play a role in TB outcomes affecting women living with HIV. Employment is a determinant for economic stability and opportunity; however, it is often explored as related to TB apart from the examination of discrimination. Individuals who may be capable, willing and able to work are denied opportunities for employment, promotion and economic stability because of their HIV status, which may influence poor health outcomes such as TB. Advanced HIV disease has been associated with an increased risk of employment loss among women but not men, highlighting the need for enforceable protections, especially for women [43]. The prevalence of employment discrimination was lower in settings with non‐discrimination protections compared to settings without these protections, which may be driving the observed association between discrimination and TB diagnosis. Given this, non‐discrimination protections related to employment may support improved TB outcomes among women living with HIV. The HIV response has prioritized the elimination of stigma and discrimination as a core component of achieving HIV control, and aligning these efforts with the TB response may contribute to TB control [1, 8, 24, 44].

Several limitations should be considered in the interpretation of this study. TB diagnosis may represent the development of TB disease and subsequent diagnosis, or conversely, it may represent access to and engagement in care and early detection, making interpretation challenging. Given this, there may be selection bias in our sample, which may disproportionately represent those individuals engaged in care, and may not accurately represent the development of TB disease among individuals not engaged in care. Selection bias may have also occurred related to experiences of employment and discrimination. Additionally, self‐reported TB diagnosis may be subject to recall bias, in which the participant does not accurately report a diagnosis, or social desirability bias, in which the participant may not report a diagnosis to the study interviewer. Lastly, medical records were not available to use as a validation method for TB diagnosis. Employment discrimination was reported as based on HIV status; however, if an individual experienced discrimination for other reasons, this was not captured in these data. There are large informal economies in many countries and, therefore, employment in this context may be more nuanced as engagement in the informal or formal economy. Data linking sampling approaches to individual participants was not available and, therefore, not included in the analysis, which may introduce bias to the estimates obtained. When assessing EMM of the presence of policies, we considered a qualitative change in results to suggest potential differences in contexts, even though the interaction terms to confirm statistically significant findings were not observed. This may be due to the small number of countries included in these data, or it may also be difficult to differentiate two specific policies from other country‐level (policy or non‐policy‐related) factors. Additionally, there may be unmeasured confounding at the country level. For example, employment instability as well as TB outcomes may also be affected by factors such as political instability, conflict or climate events, which were not accounted for in this study. Data on TB preventive treatment among participants was not available in these data, which may serve an important role in interpreting these results. For example, women who received TB preventive treatment may not have been diagnosed with active TB—not because they were never at risk, but because the preventive therapy effectively reduced their likelihood of developing the disease. Importantly, this study includes survey data from cross‐sectional studies, and, therefore, this study cannot assess causality. We posit that the associations employment discrimination may precede the development of TB disease, and that they may be causally linked. However, it is possible that this association is bi‐directional, and that development of TB disease may precede employment discrimination overall or for some individuals. Prospective, longitudinal data would help to clarify the associations explored in these analyses.

## 5 CONCLUSIONS

Poverty is a complex, structural, multi‐sectoral and multi‐factor issue which remains determinantal across health and development sectors. Our study demonstrates that the presence of social protections modifies the associations between economic instability and employment discrimination, and TB diagnosis. Given the role of poverty in driving TB epidemics, social protections focused on employment, economic instability and opportunity may support TB prevention and control. However, policy alone is not sufficient to overcome economic barriers and experiences of discrimination, and their potentially deleterious effect on TB. Establishment and enforcement of non‐discrimination protections along interventions across socio‐ecological levels and sectors may improve economic instability and support TB outcomes among women living with HIV. Lastly, examination of the causal relationship between employment discrimination and TB would provide clarity and insight into the effect of employment discrimination on TB.

## COMPETING INTERESTS

There are no conflicts of interest.

## AUTHOR CONTRIBUTIONS

CL, GT, SDB, OS, PL, LS, CGLM, KD and SB contributed to data collection. CL and BG led the conceptualization of the study with input from CB, OS, PL, HS and SB. CL led the analysis, and OS, PL, SB, KD, FC, DO, SDB, DD, CB and BG provided feedback and input in the analytical phase. CL drafted the manuscript. All authors reviewed the manuscript and provided feedback: OS, PL, GT, JDA, SB, KD, FC, DO, LS, CGLM, HS, AR, KR, SB, RC, DD, CB and BG. All authors have read and approved the final manuscript.

## ACKNOWLEGEMENTS

We express our sincere appreciation to the participants of this study who took the time to contribute to these data. We appreciate the leadership of the networks of people living with HIV who designed, led and implemented this study in each country.

## FUNDING

The analysis and manuscript were made possible through efforts funded through NIMH, including the National Institute of Mental Health of the National Institutes of Health under Award Numbers F31MH128079 and R01MH110358. Also, through the T32 NRSA Postdoctoral Training Fellowship in HIV Epidemiology and Prevention Sciences (2T32AI102623‐08) within the Johns Hopkins University Center for Public Health and Human Rights. Additional support was provided by NIH awards R01AI170249 and K01MH129226. This publication was made possible by the Center for HIV and Mental Health Stigma Elimination Strategies (P30MH136919); the Johns Hopkins University Center for AIDS Research (P30AI094189); and the Johns Hopkins Tuberculosis Research Advancement Center (P30AI168436). The funders had no role in study design, data collection and analysis, decision to publish or preparation of the manuscript.

## DISCLAIMER

The content is solely the responsibility of the authors and does not necessarily represent the official views of the National Institutes of Health. Additionally, despite the inclusion of co‐authors from the World Health Organization and UNAIDS, this manuscript does not represent the views or opinions of these organizations.

## Supporting information




**Table S1**. Summary of women living with HIV who participated in the People Living with HIV Stigma Index 2.0 study in 11 countries across sub‐Saharan Africa between 2021 and 2022.
**Table S2**. Country‐level measures for HIV and TB epidemics and responses, and data.
**Table S3**. Economic instability, and employment discrimination and associations with recent TB diagnosis among women living with HIV in 11 countries in sub‐Saharan Africa.
**Table S4**. Economic instability, and employment discrimination and associations with recent TB diagnosis among women living with HIV in 11 countries in sub‐Saharan Africa, stratified by country‐level non‐discrimination protections.

## Data Availability

The data that support the findings of this study are available from the corresponding author upon reasonable request.
