# Supplementary material for: Characterizing tuberculosis diagnosis and the associations with economic instability and employment discrimination among women living with HIV across 11 countries in sub‐Saharan Africa: a cross‐sectional study
Source: J Int AIDS Soc. 2026 Jan 6;28(12):e70022. doi: 10.1002/jia2.70022 (PMC12774796; doi:10.1002/jia2.70022)
Supplement: Supplementary file 1 — Table S1. Summary of women living with HIV who participated in the People Living with HIV Stigma Index 2.0 study in 11 countries across sub‐Saharan Africa between 2021 and 2022. Table S2. Country‐level measures for HIV and TB epidemics and responses, and data. Table S3. Economic instability, and employment discrimination and associations with recent TB diagnosis among women living with HIV in 11 countries in sub‐Saharan Africa. Table S4. Economic instability, and employment discrimination and associations with recent TB diagnosis among women living with HIV in 11 countries in sub‐Saharan Africa, stratified by country‐level non‐discrimination protections. [file JIA2-28-e70022-s001.docx]

**SUPPLEMENTARY TABLES**

Supplementary Table 1. Summary of women living with HIV who participated in the People Living with HIV Stigma Index 2.0 study in 11 countries across sub-Saharan Africa between 2021 and 2022.

Supplementary Table 2. Country level measures for HIV and TB epidemics and responses, and data.

Supplementary Table 3. Economic instability, and employment discrimination and associations with recent TB diagnosis among women living with HIV in 11 countries in sub-Saharan Africa.

Supplementary Table 4. Economic instability and employment discrimination and associations with recent TB diagnosis among women living with HIV in 11 countries in sub-Saharan Africa stratified by country level non-discrimination protections.

# SUPPLEMENTARY TABLES

**Supplementary Table 1. Summary of women living with HIV who participated in the People Living with HIV Stigma Index 2.0 study in 11 countries across sub-Saharan Africa between 2021 and 2022.**

|  | **Study sample** | | **Demographic characteristics** | | | **Recent TB diagnosis** | |
| --- | --- | --- | --- | --- | --- | --- | --- |
|  | Proportion of overall sample | | Age | Relationship | Education | No | Yes |
|  | % | n | Median (IQR) | % (Yes) | % (Yes) | % | % |
| Overall | 100 | 10718 | 39 (31-47) | 58.50 | 73.76 | 94.06 | 7.59 |
| COUNTRY |  |  |  |  |  |  |  |
| Benin | 5.41 | 580 | 39 (34-47.5) | 51.90 | 55.34 | 93.45 | 6.55 |
| Burkina Faso | 15.50 | 1661 | 42 (34-49) | 51.02 | 49.22 | 92.17 | 7.83 |
| Cote d’Ivoire | 14.20 | 1522 | 39 (31-47) | 59.46 | 52.63 | 91.91 | 8.09 |
| Ghana | 12.28 | 1316 | 41 (33-49) | 43.31 | 74.39 | 93.31 | 6.69 |
| Kenya | 11.77 | 1261 | 38 (30-45) | 64.16 | 91.20 | 91.67 | 8.33 |
| Mauritania | 3.69 | 396 | 41 (35-49) | 40.66 | 63.38 | 85.89 | 14.11 |
| Togo | 6.88 | 737 | 40 (33-48) | 52.37 | 73.95 | 95.12 | 4.88 |
| Zimbabwe | 6.73 | 721 | 40 (31-50) | 66.16 | 96.26 | 94.45 | 5.55 |
| Angola | 8.54 | 915 | 34 (29-41) | 66.01 | 87.54 | 90.49 | 9.51 |
| Lesotho | 7.63 | 818 | 36 (29-44) | 78.85 | 96.09 | 91.66 | 8.34 |
| Nigeria | 7.38 | 791 | 38 (32-44) | 71.36 | 96.08 | 94.06 | 5.94 |

Supplementary Table 2. Country level measures for HIV and TB epidemics and responses, and data.

|  | **HIV and TB epidemics and responses** | | | | **Data** | |
| --- | --- | --- | --- | --- | --- | --- |
|  | **HIV prevalence among adults 15-49** | **Total TB incidence (Rate per**  **100 000 population)** | **Funding for TB, 2021 (US$ millions)** | **TB treatment coverage (notified/estimated incidence), 2021** | **Non-discrimination protections policy status** | **Year of data collection People Living with HIV Stigma Index 2.0** |
| **COUNTRY** |  |  |  |  |  |  |
| Benin | 0.8% | 53 (33-77) | 3,000,000 | 54% (37-86) | Not adopted | 2021 |
| Burkina Faso | 0.6% | 45 (29-65) | 2,000,000 | 69% (48-110) | Not adopted | 2022 |
| Cote d’Ivoire | 1.9% | 128 (80-187) | 21,000,000 | 59% (40-95) | Adopted | 2020 |
| Ghana | 1.7% | 136 (63-237) | 8,000,000 | 30% (17-65) | Adopted | 2020 |
| Kenya | 4.0% | 251 (152-373) | 30,000,000 | 57% (38-94) | Not adopted | 2021 |
| Mauritania | 0.3% | 81 (51-119) | 0 | 65% (44-100) | Not adopted | 2022 |
| Togo | 1.9% | 33 (26-41) | 2,000,000 | 84% (68-110) | Not adopted | 2020 |
| Zimbabwe | 11.6% | 190 (135-253) | 21,000,000 | 54% (40-75) | Adopted | 2022 |
| Angola | 1.6% | 325 (213-459) | 8,000,000 | 55% (39-84) | Adopted | 2022 |
| Lesotho | 20.9% | 614 (382-900) | 11,000,000 | 32% (22-52) | Not adopted | 2020 |
| Nigeria | 1.3% | 219 (143-311) | 0 | 44% (31-67) | Not adopted | 2021 |

**Supplementary Table 3. Economic instability, and employment discrimination and associations with recent TB diagnosis among women living with HIV in 11 countries in sub-Saharan Africa.**

|  | **Recent TB diagnosis** | | | | | |
| --- | --- | --- | --- | --- | --- | --- |
|  |  |  | **Unadjusted model** | | **Adjusted for level 1 and 2 variables** | |
|  | **N** | **%** | **OR** | **95% CI** | **AOR*** | **95% CI** |
| **Economic instability** |  |  |  |  |  |  |
| Employment (any) | 6281 | 58.61 | 0.69 | 0.59, 0.79 | 0.72 | 0.61, 0.84 |
| Unable to meet basic needs (food, shelter, or clothing) |  |  |  |  |  |  |
| Never | 2096 | 19.56 | Ref | Ref | Ref | Ref |
| Sometimes | 5951 | 55.53 | 1.14 | 0.93, 1.39 | 1.07 | 0.87, 1.32 |
| Most of the time | 2669 | 24.91 | 1.53 | 1.23, 1.90 | 1.34 | 1.06, 1,69 |
| **Employment discrimination** |  |  |  |  |  |  |
| Ever refused employment or income due to HIV status | 624 | 6.70 | 1.71 | 1.33, 2.22 | 1.74 | 1.32, 2.30 |
| Ever refused promotion due to HIV status | 438 | 5.14 | 1.93 | 1.43, 2.59 | 1.84 | 1.33, 2.53 |

Tuberculosis (TB); Odds ratio (OR); Adjusted odds ratio (OR); antiretroviral therapy (ART)

* Logistic regression models adjusted for age, education, relationship, time from HIV diagnosis, current ART use, time from diagnosis to treatment initiation, HIV prevalence among adults, HIV prevalence among women, TB incidence, TB funding, TB treatment coverage

**Supplementary Table 4. Economic instability and employment discrimination and associations with recent TB diagnosis among women living with HIV in 11 countries in sub-Saharan Africa stratified by country level non-discrimination protections.**

|  | **Lack of Non-discrimination protections** | | | | | | **Presence of Non-discrimination protections** | | | | | |  |
| --- | --- | --- | --- | --- | --- | --- | --- | --- | --- | --- | --- | --- | --- |
|  | **Total (N=6244)** | | **Recent TB diagnosis (N=469)** | | | | **Total (N=4311)** | | **Recent TB diagnosis (N=338)** | | | | **Interaction**** |
|  | **%** | **N** | **%** | **N** | **AOR*** | **95% CI** | **%** | **N** | **%** | **N** | **AOR*** | **95% CI** | **p-value** |
| **Economic instability** |  |  |  |  |  |  |  |  |  |  |  |  |  |
| Employment (any) | 57.52 | 3591 | 49.68 | 233 | 0.72 | 0.59, 0.88 | 60.13 | 2690 | 50.89 | 172 | 0.72 | 0.56, 0.92 | 0.912 |
| Unable to meet basic needs |  |  |  |  |  |  |  |  |  |  |  |  |  |
| Never | 19.34 | 1207 | 16.84 | 79 | Ref | Ref | 19.87 | 889 | 15.98 | 54 | Ref | Ref | Ref |
| Sometimes | 55.64 | 3473 | 55.44 | 260 | 1.16 | 0.89, 1.53 | 55.39 | 2478 | 48.52 | 164 | 0.99 | 0.70, 1.39 | 0.674 |
| Most of the time | 25.02 | 1562 | 27.72 | 130 | 1.14 | 0.83, 1.57 | 24.74 | 1107 | 35.50 | 120 | 1.63 | 1.14, 2.36 | 0.044 |
| **Employment discrimination** |  |  |  |  |  |  |  |  |  |  |  |  |  |
| Ever refused employment or income due to HIV status | 8.16 | 444 | 13.72 | 55 | 1.86 | 1.34, 2.59 | 4.65 | 180 | 6.05 | 17 | 1.52 | 0.87, 2.63 | 0.495 |
| Ever refused promotion due to HIV status | 6.57 | 319 | 12.50 | 43 | 2.00 | 1.37, 2.91 | 3.25 | 119 | 4.43 | 12 | 1.50 | 0.79, 2.89 | 0.398 |

Tuberculosis (TB); Odds ratio (OR); Adjusted odds ratio (OR); antiretroviral therapy (ART)

******* *Logistic regression models adjusted for age, education, relationship, time from HIV diagnosis, consistent ART use, HIV prevalence among adults, TB incidence, TB funding, TB treatment coverage.*

***As a sensitivity analysis, we included an interaction term between our exposure of interest and non-discrimination policy to the model to assess if the association was statistically significantly different between the policy environments.*
